# Risk of Second Malignant Neoplasm in Familial Non-Medullary Thyroid Cancer Patients

**DOI:** 10.3389/fendo.2022.845954

**Published:** 2022-03-04

**Authors:** Marco Capezzone, Alfonso Sagnella, Silvia Cantara, Noemi Fralassi, Fabio Maino, Raffaella Forleo, Lucia Brilli, Tania Pilli, Alessandra Cartocci, Maria Grazia Castagna

**Affiliations:** ^1^ Unit of Endocrinology, Misericordia Hospital, Grosseto, Italy; ^2^ Department of Medical, Surgical and Neurological Sciences, University of Siena, Siena, Italy; ^3^ Department of Medical Biotechnologies, University of Siena, Siena, Italy

**Keywords:** thyroid cancer, second malignant neoplasm, sporadic NMTC, familial NMTC, differentiated thyroid cancer

## Abstract

**Introduction:**

Survival rates in patients with non-medullary thyroid carcinoma (NMTC) are high, increasing the possibility to develop a second malignant neoplasm (SMN). Many studies investigated the relationship between increased risk of SMN in NMTC patients treated with radioiodine, but few data are available about the impact of family history (FH) of thyroid cancer on SMN risk.

**Purpose:**

To assess the risk of SMN in a large cohort of sporadic and familial NMTC using the standardized incidence ratio (SIR).

**Patients and methods:**

We studied 918 NMTC patients (73.9% female patients) followed for a median follow-up of 9 years. In 798/918 (86.9%) patients, NMTC was sporadic, while the remaining 120 (13.1%) were familial NMTC (FNMTC).

**Results:**

We identified 119/918 (13%) patients with SMN in association with NMTC. NMTCs had an increased risk of SMN when compared to the general population (SIR 2.1, 95% CI 1.7–2.5). The rate of SMN for all sites was significantly higher in familial compared to sporadic NMTC (20% versus 11.9%, *p* = 0.01), primarily driven by families with more than two affected members. The risk of SMN was remarkably higher for breast cancer, especially in familial cases (SIR 22.03, 95% CI 14.4–41.2) compared to sporadic cases (SIR:17, 95% CI 11.9–24.6).

**Conclusions:**

NMTC patients have a higher risk of SMN compared to the general population and this risk is much higher in patients with FNMTC. This observation raises the hypothesis that genetic risk factors for a first cancer may predispose to SMN, especially among individuals with familial clustering of the same or other tumors.

## Introduction

Non-medullary thyroid carcinoma (NMTC), and in particular the papillary histotype (PTC), is the most common endocrine tumor, and it is responsible for 567,000 cases/year worldwide, ranking ninth for incidence (1). Survival rate of NMTC has improved over time, leading to the question if the lifetime risk of developing a second malignant neoplasm (SMN), usually defined as primary malignant tumors of different histological origins in one person, may be increased. To date, some studies showed a higher risk of SMN in NMTC patients compared with the general population related to 131-I therapy (2–4). Nevertheless, other studies suggested that the higher incidence of SMN could be explained by common environmental or genetic risk factors (5, 6). Family history (FH) is a known risk factor for many cancers, since about 50% of cancer patients have a first-degree relative diagnosed with some other (discordant) cancers. The impact of family history has been demonstrated in Hodgkin lymphoma where an increased risk of a second cancer (i.e., lung, colorectal, and breast cancers) was found in survivors with a first-degree relative with cancer (7). Similar observations have also been reported for melanoma, prostate, and breast cancers (8–10). Moreover, a family history of cancer has been advocated as a risk factor for other common malignancies in NMTC, due to shared genetic background (10).

Familial non-medullary thyroid cancer (FNMTC), defined by the presence of the tumor in two or more first-degree relatives, in the absence of other predisposing hereditary causes, is reported in nearly 10% of NMTC patients (11). However, this definition is still controversial. Some authors consider kindred with only two affected members a fortuitous association, whereas only families with 3 or more affected first-degree relatives should be considered FNMTC. Recently, our group suggested that the definition of FNMTC in kindred with only 2 affected members should also take into account the age at diagnosis as a key element of familial cancer (12). There are still many unclear aspects concerning definition, clinical behavior, and genetic mechanisms underlying this type of thyroid cancer (13, 14). Recently, some new insights into genetics of FNMTC have emerged from the recent genome-wide association studies (GWAS) and several single-nucleotide polymorphisms (SNPs) have been reported (15, 16). A family history of thyroid cancer is a well-established risk factor for NMTC, and it has been suggested that it may be associated with an increased risk for other primary cancers (17). Nevertheless, to our knowledge, there are no studies evaluating the impact of FNMTC on the incidence of SMN in patients with NMTC.

Therefore, this study was designed (1) to assess the risk of SMN in a large cohort of NMTC using the standardized incidence ratio (SIR) and (2) to verify the role of family history of thyroid cancer in the occurrence of SMN.

## Patients and Methods

### Study Population

An observational single-center study was conducted on 918 NMTC patients followed at the Section of Endocrinology, University of Siena (Italy), from 1995 to 2015. Patients with prior exposure to radiation and with anaplastic thyroid carcinoma, medullary thyroid carcinoma, malignant lymphoma, or other inherited familial cancer syndromes (e.g., familial adenomatous polyposis, Gardner’s syndrome, Cowden’s disease, Carney’s complex, and Werner’s syndrome) were excluded. Surgical, pathological, and clinical data were retrieved from clinical records. Patients gave their informed consent to the use of their personal data for research purposes, and the study was approved by the local ethical committee.

### Non-Medullary Thyroid Carcinoma Treatment Modalities and Follow-Up

Initial treatment and follow-up strategy were based on the therapeutic protocols used before the introduction of the latest 2016 ATA guidelines. Almost all patients were submitted to near-total thyroidectomy followed by 131-I treatment. Lymphadenectomy was performed if suspicious lymph node metastases were found before surgery. Patients were followed every 6 months during the first year; subsequently, the frequency of the follow-up visits was based on the clinical course of the disease and the estimated risk of recurrence for each patient. At each control, basal or stimulated serum thyroglobulin (Tg), thyroglobulin antibodies (AbTg), and neck ultrasound were performed. Additional imaging procedures, such as 18FDG-PET/CT, RMN, computed tomography (CT) scan of the chest, and post-therapeutic 131-I whole body scan, were performed in selected patients to exclude the presence of distant metastases. Response to the initial therapy and the clinical status at each follow-up visit was defined according to the risk stratification system recommended by the 2016 ATA guidelines (18). According to our clinical protocol, treatment and follow-up were the same for familial and sporadic NMTC.

### Definitions of Second Malignant Neoplasm

Second Malignant Neoplasm (SMN) was defined as any primary malignancy with histological confirmation occurring in an anatomical site other than the thyroid. According to the timing of SMN occurrence with respect to NMTC diagnosis, we divided the SMN group into pre-NMTC (not metachronous) and post-NMTC (metachronous), based on the tumor diagnosis made at least 12 months before or after NMTC, respectively. The SMNs diagnosed within this period were considered synchronous.

### Statistical Analysis

Statistical analysis was performed using the software StatView for Windows version 5.0.1 (SAS Institute, Cary, NC) and the *R* version 3.6.2. Data were presented as mean ± SD or median when needed or as absolute frequencies and percentages. The *T* test for independent data or the Mann–Whitney *U* test was performed for variables, normally or non-normally distributed (evaluated by Kolmogorov–Smirnov), respectively. To evaluate significant differences in data frequency, the Chi-squared test, the Fisher exact test, or its approximation was performed, according to the dimension of contingency table and to the expected frequencies. The comparison between the Italian cancer risk and the risk of SMN in patients with thyroidal cancer diagnosed before and after NMTC was estimated using standardized incidence rate (SIR), which is a ratio of an observed to an expected number of patients with SMN. The expected number of SMN during the person‐years at risk was determined on the basis of gender, age, and calendar year specific incidence rates from the Italian Network of Cancer Registries (AIRTUM) data from 1976 and 2010, which includes 38 general cancer registries, covering almost 48% of the Italian population (19). Relative risk (RR) and the relative 95% confidence interval (CI) were estimated. Stepwise log-binomial regression was performed to evaluate the adjusted RRs and their confidence interval. A *p*-value <0.05 was considered statistically significant (20).

## Results

### Clinical and Pathological Features of the Study Group

As shown in **Table 1**, the study population included 678 (73.8%) female patients and 240 (26.2%) male patients (F/M:3/1), with a median age of 46 years (range, 7-87 years). Most patients (897/918, 97.7%) were affected by PTC, and the remaining 21 patients (2.3%) had a follicular histotype (FTC). Among this series, we identified 120 (13.1%) patients with a positive family history that were classified FNMTC because at least one first-degree relative was affected by confirmed thyroidal cancer of follicular origin. The remaining 798/918 NMTC patients (86.9%) were sporadic. Almost all patients (906/918, 98.7%) were submitted to total thyroidectomy with or without lymphadenectomy. Radioiodine therapy was performed in 720/906 patients (79.5%). At final histology, minimal extrathyroidal extension was found in 336/918 patients (36.6%), while lymph node metastases were observed in 248/918 cases (27%). Forty-six (5%) patients presented distant metastases at diagnosis. At final follow-up, 717/918 (78.1%) patients had an excellent response, and 148/918 (16.1%) had structural incomplete response. Thyroid cancer-related death occurred in 12/918 (1.3%), while 41/918 (4.5%) patients were lost at follow-up. Mean follow-up was 10 ± 4.8 years (median 9 years).

**Table 1:** Clinical, pathological, and epidemiological features of our study group.

Parameters	Number of patients (%) (*n* = 918)
**Gender: n (%)**	
Male	240 (26.2)
Female	678 (73.8)
**Age at diagnosis: (years)**	
Mean ± SD	46.8 ± 16.1
Range	7–87
Median	46
**Cancer histotypes: *n* (%)**	
Papillary	897 (97.7)
Follicular	21 (2.3)
**Type of NMTC: *n* (%)**	
Sporadic	798 (86.9)
Familial	120 (13.1)
**Type of surgery: *n* (%)**	
Total thyroidectomy	906 (98.7)
Hemithyroidectomy	12 (1.3)
**Radioiodine treatment: *n* (%)**	
Yes	720 (79.5)
**Tumor extension: *n* (%)**	
Intrathyroidal	582 (63.4)
Extrathyroidal #	336 (36.6)
**Lymph-node metastases: *n* (%)**	
Yes	248 (27)
Not	670 (73)
**Distant metastases: *n* (%)**	
Yes	46 (5)
Not	872 (95)
**Disease status: *n* (%)**	
Excellent response	717 (78.1)
Lost at follow-up	41 (4.5)
Structural incomplete and indeterminate response	148 (16.1)
Thyroid cancer-related death	12 (1.3)
**Follow-up: (years)**	
Mean ± SD	10 ± 4.8
Range	5–47
Median	9

^#^Minimal extrathyroid extension (e.g., perithyroidal soft tissues or sternothyroid muscle) from a tumor of any size (TNM-7th Edition).

### Second Malignant Neoplasm in Non-Medullary Thyroid Cancer Patients

**Figure 1:**
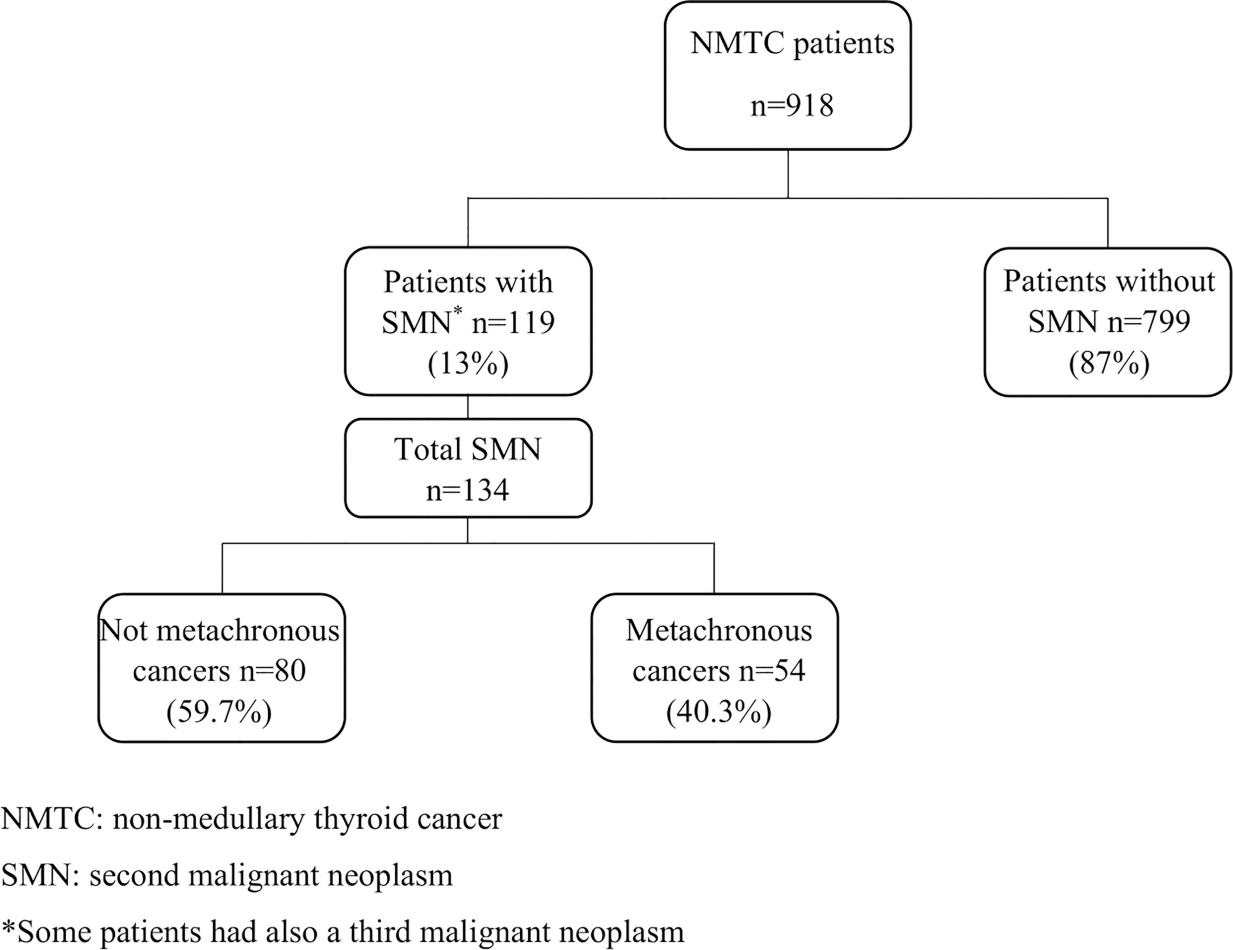
Study population description.

As illustrated in **Figure 1**, 119/918 (13%) NMTC patients were affected by other malignancies in association with thyroid cancer. Moreover, in 15/119 (12.6%) patients, a third primary malignancy was also observed during follow-up. Fifty-four/134 (40.3%) patients with SMN were metachronous, while the remaining 80 cases (69.7%) of SMNs were diagnosed before the thyroid cancer diagnosis (**Table 2**).

**Table 2:** Second malignant neoplasms (SMNs) observed before and after radioiodine treatment.

Parameters	SMNs before I-131	SMNs after I-131
Number of patients (%)	80 (59.7)	54 (40.3)
Radioiodine administered (Mbq)		
Mean ± SD	5,827.5 ± 8,931.8	8,613.6 ± 10,208.3
Median	2,534.5	3,700
Range	1,110–38,628	110–46,324

**Figure 2:**
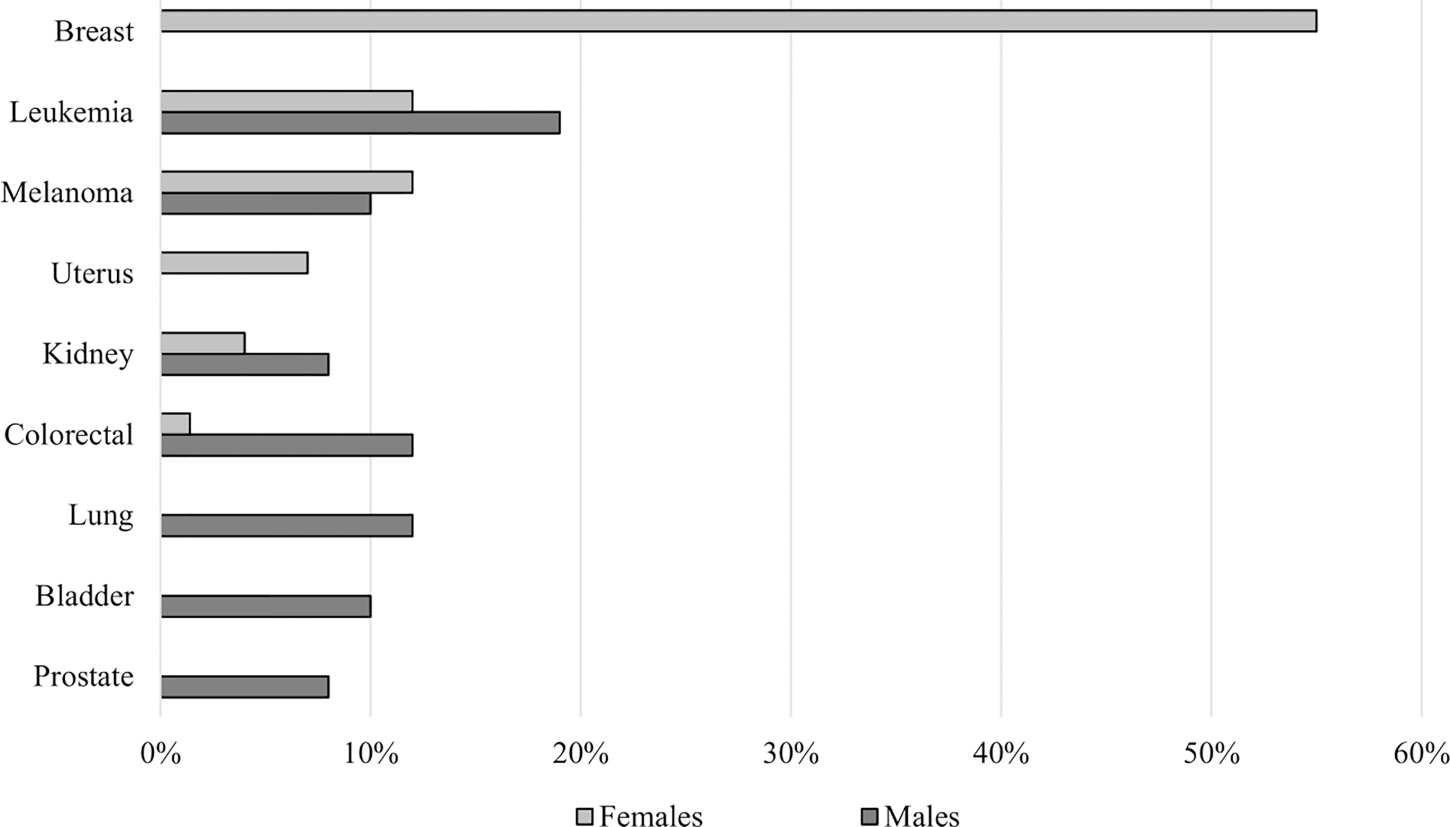
Site distribution and prevalence of SMN according to gender.

As shown in **Figure 2**, the most represented sites of SMN in female patients were breast (55%), blood (12%), skin (12%), and uterus (7%) while blood (19%), colorectal (12%), lung (12%), and skin cancer (10%) showed a higher prevalence in the male population.

As reported in **Table 3**, patients with SMN were older (*p <* 0.0001), were more frequently male (*p* = 0.0003), and had a higher rate of distant metastases at diagnosis (*p* = 0.001) when compared with patients without SMN. Patients with SMN showed the worst final outcome (*p* = 0.006) and had more frequently familial history of any type of cancer (*p* = 0.0004) and familial NMTC (*p* = 0.01). No differences were found about tumor histotype (*p* = 0.7), multicentricity (*p* = 0.6), bilaterality (*p* = 0.5), rate of lymph-node metastases (*p* = 0.8), tumor extension (*p* = 0.8), radioiodine treatment (*p* = 0.2), and follow-up length (*p* = 0.9).

**Table 3:** Clinical, pathological, and epidemiological features of NMTC patients with and without SMN.

Parameters	Group I (with SMN) (*n* = 119)	Group II (without SMN) (*n* = 799)	*p-*value
Gender: *n* (%) **			**0.0003**
Male	48 (40.3)	192 (24)	
Female	71 (59.7)	607 (76)	
Age at diagnosis: (years)*			**<0.0001**
Mean ± SD	55.6 ± 15.4	45.5 ± 15.8	
Range	15-84	7-87	
Median	56	44	
Cancer histotypes: *n* (%) **			0.7
Papillary	116 (97.5)	781 (97.7)	
Follicular	3 (2.5)	18 (2.3)	
Multifocality: *n* (%) **			0.6
Yes	45 (37.8)	320 (40.1)	
Not	74 (62.2)	479 (59.9)	
**Type of NMTC°:**			**0.01**
Sporadic	95 (79.8)	703 (88)	
Familial	24 (20.2)	96 (12)	
Bilaterality: *n* (%) **			0.5
Yes	32 (26.9)	236 (29.5)	
Not	87 (73.1)	563 (70.5)	
Tumor extension: *n* (%) **			0.8
Intrathyroidal	74 (62.2)	508 (63.6)	
Extrathyroidal #	45 (37.8)	291 (36.4)	
Lymph-node metastases: *n* (%) **			0.8
Yes	31 (26.1)	217 (27.2)	
Not	88 (73.9)	582 (72.8)	
Distant metastases: *n* (%) **			**0.001**
Yes	12 (10.1)	34 (4.3)	
Not	107 (89.9)	765 (95.7)	
Radioiodine treatment: *n* (%) **			0.2
Yes	103 (86.6)	650 (81.4)	
Not	16 (13.4)	149 (18.6)	
**Familiar history of cancer: *n* (%) ##**			**0.0004**
Yes	64 (57.1)	303 (39.2)	
No	48 (42.9)	470 (60.8)	
Follow-up (years) *			0.9
Mean ± SD	9.9 ± 4.6	10.1 ± 4.9	
Range	5-21	5-47	
Median	9	9	
Outcome: *n* (%) **			**0.006**
Excellent response	83 (69.7)	634 (79.3)	
Structural diseases	14 (11.8)	61 (7.7)	
Biochemical/indeterminate response	9 (7.6)	64 (8.0)	
Thyroid cancer-related death	5 (4.2)	7 (0.9)	
Lost at follow-up	8 (6.7)	33 (4.1)	

*By Mann–Whitney test; **By *χ* ^2^ test

# Minimal extrathyroidal extension (e.g., perithyroidal soft tissues or sternothyroid muscle) from a tumor of any size (TNM-7^th^ Edition).

## Data available in Group 1, n = 112; data available in Group 2, n = 773.

°NMTC, non-medullary thyroid cancer.

Bold means statistically significant parameters.

At multivariate analysis, male gender, familial history of any type of cancer, higher age at diagnosis, and familial NMTC were independently associated with the risk to develop SMN, yielding a relative risk (RR) of 1.54 (1.10–2.15; *p* = 0.01), 1.51 (IC 1.07–2.12; *p* = 0.02), 1.03 (IC 1.02–1.04; *p <* 0.001), and 1.75 (IC 1.20–2.54; *p* = 0.003), respectively. Moreover, patients with FNMTC had a significantly higher risk for SMN associated to thyroid cancer (**Table 4**).

**Table 4:** Multivariate logistic regression analysis for SMN prognostic factors in NMTC.

Predictor	*p*-value	Relative Risk	95% CI
Male gender	0.01	1.54	1.10–2.15
Familial history of other cancers	0.02	1.51	1.07–2.12
Age at diagnosis of NMTC*	<0.001	1.03	1.02–1.04
FNMTC**	0.003	1.75	1.20–2.54

*NMTC, non-medullary thyroid cancer.

**FNMTC, familial non-medullary thyroid cancer.

To minimize potential selection bias due to higher age at diagnosis in the group of patients with SMN, we performed propensity score matching procedure, with a ratio of 1:1. After matching, only sex and FNMTC were significantly associated with the increased risk of SMN at multivariate analysis with a RR of 1.37 (95% CI 1.06–1.75, *p* = 0.01) and of 1.71 (95% CI 1.21–2.61, *p* = 0.005), respectively.

### Second Malignant Neoplasm in Familial Non-Medullary Thyroid Carcinoma

**Figure 3:**
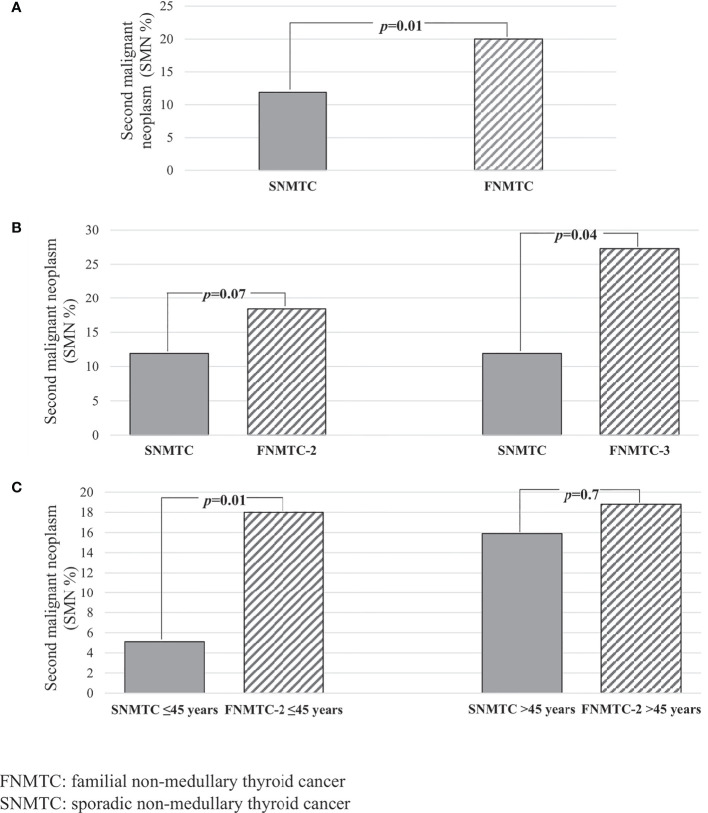
**(A)** Prevalence of SMN according to the sporadic and familial form of NMTC; **(B)** prevalence of SMN according to the number of affected members in FNMTC; **(C)** prevalence of SMN according to the age at diagnosis in FNMTC with only two family members affected.

**Figure 4:**
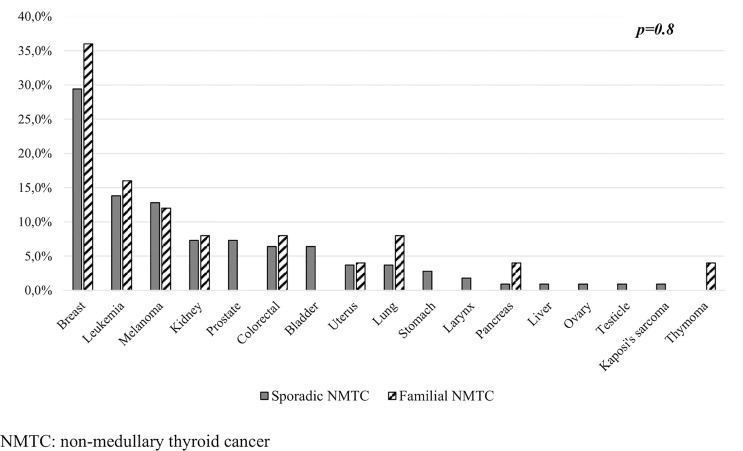
Site distribution and prevalence of SMN according to the sporadic and familial form of NMTC.

We identified 120 (13.1%) familial NMTC patients, of whom 98 (81.7%) belong to kindreds with two affected members (fNMTC-2) and 22 (18.3%) to kindreds with three or more affected members (fNMTC-3). A significantly higher rate of other primary cancers in the familial NMTC (24/120 patients, 20%) compared to sporadic NMTC (95/798 patients, 11.9%, *p* = 0.01) was found (**Figure 3A**). We observed that the rate of a second primary cancer was significantly higher in familial NMTC patients belonging to kindreds with three or more affected members compared to sporadic patients [6 (27.2%) versus 95 (11.9%) *p* = 0.04] (**Figure 3B**). On the contrary, the rate of a second cancer was not significantly different in the 98 FNMTC patients belonging to kindreds with only 2 affected members compared to sporadic NMTC patients [18 (18.4%) vs. 95 (11.9%), *p* = 0.07] (**Figure 3B**). Moreover, when the same analysis was performed according to age at diagnosis (≤45 vs. >45 years), the rate of a second primary cancer was significantly higher [14 (20.3%) vs. 15 (5.1%), *p* = 0.01] (**Figure 3C**) in familial NMTC patients with both affected members younger or equal to 45 years, while it was similar to sporadic NMTC [10 (19.6%) vs. 80 (15.9%), *p* = 0.7] (**Figure 3C**) in familial NMTC patients with at least one member affected older than 45 years. No significant difference (*p* = 0.8) in the site of SMN was observed between sporadic and familial NMTC patients (**Figure 4**).

### Standardized Incidence of SMN According to the Sporadic or Familial Presentation of NMTC

Patients with NMTC showed a higher risk of SMN compared to the general population (SIR, 2.1; 95% CI, 1.7–2.5, **Table 5**). The risk of SMN in patients with sporadic NMTC (SIR, 1.93;95% CI, 1.6–2.4) was lower (*p* = 0.04) than observed in familial NMTC (SIR, 3.0; 95% CI, 1.9–4.5). Among patients with familial NMTC, a higher SIR was observed in kindreds with three or more affected members (SIR 3.50; 95% CI, 1.29–7.62), compared to FNMTC with only two affected members (SIR 2.87; 95% CI, 1.67-4.59), but this difference was not statistically significant (*p* = 0.50). According to the site of the second primary neoplasm, a very high SIR was observed for breast cancer (SIR, 18; 95% CI, 13.1–24.7; **Table 5**), and it was higher in familial NMTC (SIR 22.0, 95% CI 14.4-41.2) than in sporadic cases (SIR 17, 95% CI 11.9–24.6), and this difference was not statistically significant (*p* = 0.50).

**Table 5:** Standardized incidence ratio of SMNs pre- and post-NMTC.

Comparison of the incidence of second primary cancer	Standardized incidence ratio	95% Confidence interval
NMTC patients	2.1	1.7–2.5
versus general population		
SNMTC patients	1.9	1.6–2.4
FNMTC patients	3.0	1.9–4.5
FNMTC-2 patients	2.8	1.6–4.5
FNMTC-3 patients	3.5	1.2–7.6
versus general population		
Breast cancer	18.2	13.1–24.7
SNMTC patients	17.4	11.9–24.6
FNMTC patients	22.0	15.4–41.2
versus general population		

SNMTC, sporadic non-medullary thyroid cancer.

FNMTC, familial non-medullary thyroid cancer.

FNMTC-2, kindreds with two affected members.

FNMTC-3, kindreds with three affected members.

## Discussion

Survival rates following cancer diagnosis have significantly improved with a resulting increase in occurrence of multiple primary cancers (21). To date, about 19% of the cancer diagnosis is made in patients with a history of a previous malignancy; therefore, the definition of the second cancer etiology is relevant for both public health and clinical practice (22). The development of multiple cancers may represent the late sequelae of the cancer treatment and/or the result of gene–environment and gene–gene interactions (23). Accordingly with the rate reported in the literature (4, 24), 13% of patients with NMTC presented a SMN in our study population. More than a half of SMN was diagnosed before the NMTC diagnosis, and, for most sites, the rate of SMN was higher than, or equal to, that diagnosed after NMTC. This type of bidirectional association between cancers raises the hypothesis of the existence of common genetic or environmental risk factors rather than a carcinogenetic effect of radioiodine therapy (25, 26). Moreover, although our study population of NMTC patients represents a slightly radioiodine-overtreated cohort prior to the 2016 ATA guidelines, SMN was developed after radioiodine therapy in only 40% of cases (**Table 2**), suggesting that radioiodine may have played a marginal role in our study population. While the role of radioiodine therapy has been expensively studied (6, 27, 28), the impact of genetic background in the cumulative risk of developing SMN in thyroid cancer patients has not been well explored. Our results seem to support the role of genetic background, since we found a higher rate of SMN in FNMTC compared to sporadic NMTC (20% versus 11.9%, *p* = 0.01). Moreover, in familial NMTC, the rate of SMN correlated with the number of affected members and the risk of SMN was primarily driven by families with more than two members. Following our recent paper (12), we evaluated the rate of SMN, in FNMTC kindreds with two affected members, considering the age at diagnosis. In familial NMTC kindreds in which age at diagnosis was equal to or less than 45 years in both affected members, the rate of SMN was significantly higher than observed in sporadic NMTC (20.3% vs. 5.3%, *p* = 0.01). Our results show that patients with NMTC have a higher risk in developing SMN when compared with the general population and demonstrate, for the first time, that this risk was higher in familial than sporadic NMTCs (SIR 3.0, 95% CI 1.9–4.5 vs. SIR 1.9, 95% CI 1.6–2.4). A possible explanation of the increased risk for FNMTC patients may be the effect of complex genetic and environmental factors or a greater genomic instability of the FNMTC patients compared to sporadic counterparts. To date, a prevalent polymorphism or germline mutations have not been identified yet in FNMTC, although several studies on different populations of NMTC patients identified common variants associated to NMTC risk with additive effects on cancer predisposition (29, 30).

We observed that the most common site for the second malignancy was breast, accounting for 34.4% of all SMNs. We found a higher risk for breast cancer (BC) in FNMTC patients (SIR 22.03, 95% CI 15.4–41.2) compared to sporadic patients and the general population. A recent meta-analysis suggested that the risk of developing thyroid cancer as a second primary malignancy in the context of BC and *vice versa* is increased compared to other SMN (31). In our NMTC patients, the number of patients diagnosed with BC before and after thyroid cancer occurrence was similar and we might hypothesize that shared risk factors may account for this association.

The impact of familial NMTC in increasing the rate of SMN has also been confirmed by multivariate analysis in which family history of other tumors, male gender, and age at diagnosis, in addition to the familial form of NMTC, were independently associated with a higher risk of SMN. Family history of other tumors is a well-recognized risk factor for SMN in patients affected by different human cancers, even in individuals without well-established familial cancer syndromes (32). We found that male gender conferred a relative risk for SMN of 1.54 (*p* = 0.01). Accordingly, a meta-analysis showed that male gender is an independent risk factor for the association with another cancer (33). Also, NMTC age at diagnosis influenced the SMN risk, and this observation was widely reported as well (2, 25, 33).

The present study has strengths and limitations. The strengths include patients followed at the same institute with detailed information regarding NMTC diagnosis, treatment, and follow-up and occurrence of SMN before or after NMTC detection. In addition, the mean follow-up of 9 years is relatively long. A possible limitation is represented by the relatively small number of FNMTC patients with three or more affected members, but it is important to underline that this group is uncommon and accounts for less than 5% of major FNMTC series.

In conclusion, we demonstrated that the risk of SMN was higher in FNMTC patients compared to sporadic NMTC patients. Our results suggest that a family history of thyroid cancer or other tumors, along with male gender and age at diagnosis of NMTC, increased the risk of SMN in NMTC patients. We can hypothesize that different cancers share common genetic factors and that FNMTC may represent a good model for exploring the potential role of genetic determinants that, probably in association with environmental factors, contribute to increase the relative risk for the same or other cancers beyond the nuclear family. Moreover, prospective studies in larger cohorts of patients with familial NMTC are needed in order to confirm our results and to translate this information in clinical practice. In light of our results, for example, the screening for a second cancer, and especially for breast cancer, might be suggested for patients with familial NMTC.

## Data Availability Statement

The raw data supporting the conclusions of this article will be made available by the authors, without undue reservation.

## Ethics Statement

The studies involving human participants were reviewed and approved by Comitato Etico Regione Toscana – Area Vasta Sud Est. The patients/participants provided their written informed consent to participate in this study.

## Author Contributions

MarcC was the main author and contributed to conception and design of the study. AS, NF, and SC organized the database. MariC contributed to interpretation of data for the work and manuscript revision. AC performed the statistical analysis. TP, FM, RF, and LB contributed to manuscript revision, read, and approved the submitted version. All authors contributed to the article and approved the submitted version.

## Conflict of Interest

The authors declare that the research was conducted in the absence of any commercial or financial relationships that could be construed as a potential conflict of interest.

## Publisher’s Note

All claims expressed in this article are solely those of the authors and do not necessarily represent those of their affiliated organizations, or those of the publisher, the editors and the reviewers. Any product that may be evaluated in this article, or claim that may be made by its manufacturer, is not guaranteed or endorsed by the publisher.
